# Risk factors for pediatric surgical site infection following neurosurgical procedures for hydrocephalus: a retrospective single-center cohort study

**DOI:** 10.1186/s12871-021-01342-5

**Published:** 2021-04-21

**Authors:** Miho Shibamura-Fujiogi, Jennifer Ormsby, Mark Breibart, Benjamin Warf, Gregory P. Priebe, Sulpicio G. Soriano, Thomas J. Sandora, Koichi Yuki

**Affiliations:** 1 Cardiac Anesthesia Division, Department of Anesthesiology, Critical Care and Pain Medicine, Boston Children’s Hospital, 300 Longwood Avenue, Boston, MA 02115 USA; 2 Department of Anaesthesia, Harvard Medical School, Boston, USA; 3 Department of Immunology, Harvard Medical School, Boston, USA; 4 Department of Pediatrics, Division of Infectious Diseases, Boston Children’s Hospital, Boston, USA; 5 Department of Neurosurgery, Boston Children’s Hospital, Boston, USA; 6 Department of Pediatrics, Harvard Medical School, Boston, USA

**Keywords:** Surgical site infections, SSI, Shunt, Hydrocephalus

## Abstract

**Background:**

Infection is a major complication following cerebral spinal fluid (CSF) diversion procedures for hydrocephalus. However, pediatric risk factors for surgical site infection (SSI) are currently not well defined. Because a SSI prevention bundle is increasingly introduced, the purpose of this study was to evaluate risk factors associated with SSIs following CSF diversion surgeries following a SSI bundle at a single quaternary care pediatric hospital.

**Methods:**

We performed a retrospective cohort study of patients undergoing CSF diversion procedures from 2017 to 2019. SSIs were identified prospectively through continuous surveillance. We performed unadjusted logistic regression analyses and univariate analyses to determine an association between SSIs and patient demographics, comorbidities and perioperative factors to identify independent risk factors for SSI.

**Results:**

We identified a total of 558 CSF diversion procedures with an overall SSI rate of 3.4%. The SSI rates for shunt, external ventricular drain (EVD) placement, and endoscopic third ventriculostomy (ETV) were 4.3, 6.9 and 0%, respectively. Among 323 shunt operations, receipt of clindamycin as perioperative prophylaxis and presence of cardiac disease were significantly associated with SSI (O.R. 4.99, 95% C.I. 1.27–19.70, *p* = 0.02 for the former, and O.R. 7.19, 95% C.I. 1.35–38.35, *p* = 0.02 for the latter). No risk factors for SSI were identified among 72 EVD procedures.

**Conclusion:**

We identified receipt of clindamycin as perioperative prophylaxis and the presence of cardiac disease as risk factors for SSI in shunt procedures. Cefazolin is recommended as a standard antibiotic for perioperative prophylaxis. Knowing that unsubstantiated beta-lactam allergy label is a significant medical problem, efforts should be made to clarify beta-lactam allergy status to maximize the number of patients who can receive cefazolin for prophylaxis before shunt placement. Further research is needed to elucidate the mechanism by which cardiac disease may increase SSI risk after shunt procedures.

## Introduction

Hydrocephalus results from a disturbance of the normal pulsatile flow of cerebrospinal fluid (CSF), resulting in its abnormal accumulation within the cerebral ventricles. This can be alleviated in the short term by insertion of a reservoir or external ventricular drain (EVD). Long term, definitive treatment is most commonly accomplished by placement of a ventriculoperitoneal shunt (VPS) or by endoscopic third ventriculostomy (ETV) with or without choroid plexus cauterization (CPC) [1]. Although the rate of surgical site infections (SSIs) after neurosurgery is generally low, the clinical and financial consequences of SSIs after CSF diversion procedures are substantial [2]. Infections following neurosurgery can lead to significant complications such as seizures, neurological deficits, and malfunction of the shunt device [3, 4]. Therefore, the prevention of infection is a priority for managing these patients, and research over the years has identified modifiable risk factors and interventions [5–8]. However, infections still persist, and greater effort needs to be directed towards understanding risk factors in order to identify new ways to lower SSI rates. To compare what has been reported with our recent experience, we conducted an observational, retrospective study to identify factors associated with SSIs after CSF diversion procedures in a pediatric cohort in a single institution.

## Materials and methods

### Study cohort and patient background

This retrospective cohort study was approved by the Institutional Review Board (IRB) at Boston Children’s Hospital (BCH). The need of the informed consent was waived. All the study methods were carried out in accordance with institutional guidelines and regulations. We identified patients who underwent CSF diversion surgery (shunts, EVD, ETV) at BCH from January 2017 to September 2019. We included cases with either congenital or acquired hydrocephalus, including communicating and noncommunicating hydrocephalus. We also included patients with a history of brain tumor who subsequently needed CSF diversion, but excluded patients who underwent craniotomy for tumor resection at the time of CSF diversion surgery. Our SSI prevention bundle consisted of 4 elements: 1) Preoperative bath using soap or an antiseptic agent; 2) Skin preparation with chlorhexidine gluconate (CHG)-alcohol (ChloraPrep™), povidone-iodine alone, povidone-iodine plus CHG-alcohol, povidone-iodine plus alcohol, or all three antiseptics in combination; 3) Using clippers for hair removal; and 4) Perioperative antibiotic prophylaxis. Cefazolin was the standard antibiotic for surgical prophylaxis unless patients had a previous history of bacterial infections that would direct other choices, or patients were previously known to have reactions to penicillin or cephalosporins, and the dose was administered within 60 min prior to skin incision. When SSIs were suspected, wounds and/or CSF were cultured at the medical team and/or surgeon’s discretion. SSIs associated with CSF diversion procedures were identified through ongoing prospective surveillance by the Infection Prevention and Control department using National Healthcare Safety Network (NHSN) definitions from the US Centers for Disease Control and Prevention (CDC) [9]. Prospective surveillance for 90 days following an operation is done by Infection Prevention and Control. For ETV, we included SSI diagnosed within 30 days after surgery. For shunt and EVD procedures, both of which involve implants, we included SSI diagnosed within 90 days after surgery. When a patient had a repeated operation on different dates within the time frame of 90-day observation, the SSI was attributed to the most recently performed procedure according to NHSN definitions. We extracted the information on the demographics and comorbidities of the study cohort from the electronic medical records and we obtained intraoperative information such as medications, surgical duration and American Society of Anesthesiology (ASA) physical status classifications from the Anesthesia Information Management System™ (AIMS; Cerner, MO, USA) to obtain. Comorbidities were based on the International Classification of Diseases (ICD)-9 / ICD-10 codes. Congenital cardiac diseases, great vessel malformations, and cardiomyopathies were categorized as cardiac diseases. There were a total of 10 surgeons.

### Statistical analysis

We reported continuous variables as either means with standard deviation (SD) for normally distributed variables or medians and interquartile ranges (IQR) for variables without normal distribution. Normality of distribution was determined by Shapiro-Wilk test. Binary and categorical variables were reported using frequencies and percentages. We performed an unadjusted logistic regression analysis with SSI as the dependent variable. The effect was quantified by the odds ratio (OR) per 1-unit change in the predictor variable. If the outcome was zero or if logistic regression analysis for each categorical variable was not fit, we performed univariate analysis. We used Fisher’s exact test to compare proportions for categorical variables, and t-tests or Mann–Whitney U tests to compare means or medians for continuous variables. All hypothesis testing had a two-sided significance level of 0.05. All statistical analyses were conducted using Stata/MP 15.0 (StataCorp, College Station, TX, USA).

## Results

Among 558 CSF diversion procedures in 306 unique patients, 19 infections occurred, yielding an SSI rate of 3.4%. Characteristics of the study cohort are shown in Table 1. The SSI rates for shunt, EVD, and ETV were 4.3, 6.9, and 0%, respectively. The median number of days from surgery to the onset of SSI was 13.5 days (IQR: 5.5, 30), with a maximum of 89 days. The median number of days from surgery to the onset of SSI was 18.5 and 9 days for shunt and EVD, respectively.

**Table 1:** Comparison of SSIs incidence and days to SSIs onset by CSF diversion procedure

CSF diversion procedure	Total (***n*** = 558)	Shunt (***n*** = 323)	EVD (***n*** = 72)	ETV (***n*** = 163)
SSI incidence (%)	19 (3.4%)	14 (4.3%)	5 (6.9%)	0
Median interval of SSI (days)	13.5	18.5	9	–

*SSIs* surgical site infection, *CSF* cerebrospinal fluid, *EVD* external ventricular drain, *ETV* endoscopic third ventriculostomy

Table 2 shows all available variables and their association with SSIs in shunt procedures. In unadjusted logistic regression analysis, factors that were significantly associated with infection in shunt surgery were the administration of clindamycin as perioperative prophylaxis (O.R. 4.99, 95% C.I. 1.27–19.70, *p* = 0.02) and the presence of congenital heart disease (O.R. 7.19, 95% C.I. 1.35–38.35, *p* = 0.02). Cardiac diseases present among patients with SSI included single ventricle disease with palliative repair and valvulopathy with underlying connective tissue diseases. None of the perioperative factors, including the duration of procedure, surgeon, or type of skin preparation agent were associated with SSIs.

**Table 2:** Results of univariate and unadjusted logistic regression analyses for variables of case background and perioperative factors in SSIs after shunt surgery

	Total (***N*** = 323)		Case without SSI (***N*** = 309)		Case with SSI (***N*** = 14)		Unadjusted logistic regression		
	n	%	n	%	n	%	Odds ratio	95% CI	*p* -value
Female	129	39.9	212	68.6	9	64.3	1.20	0.39–3.69	0.74
Age(Month), median (IQR)	139	(33, 202)	131	(33, 198)	170	(145, 209)	1.00	1.00–1.01	0.16
Age									0.17*
< 1y	43	13.3	41	13.3	2	14.3	(base)		
1-2y	50	15.5	49	15.9	1	7.1	0.42	0.03–4.78	0.48
3-10y	60	18.6	60	19.4	0	0.0			
11-18y	123	38.1	114	36.9	9	64.3	1.62	0.33–7.80	0.55
18y-	47	14.6	45	14.6	2	14.3	0.91	0.12–6.77	0.93
Weight(kg), median (IQR)	33.6	(12.7, 58.2)	32.6	(12.4, 57.8)	49.8	(35.0, 58.6)	1.01	0.99–1.02	0.38
ASA Classification									
Class ≤ II	78	24.1	75	24.3	3	21.4	(base)		
Class ≥ III	245	75.9	234	75.7	11	78.6	1.18	0.32–4.32	0.81
Wound Class									1.00*
1: Clean	320	99.1	306	99.0	14	100.0			
2: Clean-Contaminated	3	0.9	3	1.0	0	0.0			
3: Contaminated	0	0.0	0	0.0	0	0.0			
4: Dirty/Infected	0	0.0	0	0.0	0	0.0			
Comorbidity									
Brain tumor	93	28.8	87	28.2	6	42.9	1.91	0.65–5.68	0.24
Cardiac disease	9	2.8	7	2.3	2	14.3	7.19	1.35–38.35	0.02
Gastrostomy status	63	19.5	58	18.8	5	35.7	2.40	0.78–7.44	0.13
Prematurity (GA < 37 week)	6	1.9	6	1.9	0	0.0			1.00*
Procedure									1.00*
V-P shunt	306	94.7	292	94.5	14	100.0			
V-A shunt	7	2.2	7	2.3	0	0.0			
L-P shunt	10	3.1	10	3.2	0	0.0			
Number of shunt revision, mean (SD)	1	(2,4)	2	(1,4)	3	(1,6)	1.12	0.99–1.27	0.07
Emergent surgery	198	61.3	190	61.5	8	57.1	0.84	0.28–2.47	0.74
Duration of surgery(min), median(IQR)	77	(56, 108)	77	(56, 108)	76	(60, 96)	1.00	0.99–1.01	0.92
Prophylactic antibiotics	320	99.1	306	99.0	14	100.0	–		1.00*
Cefazolin	274	84.8	264	85.4	10	71.4	0.43	0.13–1.42	0.16
Ampicillin	1	0.3	1	0.3	0	0.0			1.00*
Cefoxitin	1	0.3	1	0.3	0	0.0			1.00*
Ciprofloxacin	4	1.2	4	1.3	0	0.0			1.00*
Cefepime	5	1.5	4	1.3	1	7.1	5.87	0.61–56.2	0.13
Ceftazidime	1	0.3	1	0.3	0	0.0			1.00*
Ceftriaxone	5	1.5	5	1.6	0	0.0			1.00*
Clindamycin	19	5.9	16	5.2	3	21.4	4.99	1.27–19.70	0.02
Metronidazole	0	0.0	0	0.0	0	0.0			
Vancomycin	23	7.1	21	6.8	2	14.3	2.29	0.48–10.89	0.3
Meropenem	3	0.9	3	1.0	0	0.0			1.00*
Surgeon *n* = 10	–	–	–	–	–	–			0.54*
Skin preparation agent									0.93*
None	36	11.1	35	11.3	1	7.1	(base)		
CHG-alcohol(Chloraprep™)	235	72.8	224	72.5	11	78.6	1.72	0.22–13.72	0.61
Povidone-iodine	17	5.3	16	5.2	1	7.1	2.19	0.13–37.22	0.59
Povidone-iodine and CHG-alcohol	5	1.5	5	1.6	0	0.0			
Povidone-iodine and alcohol	28	8.7	27	8.7	1	7.1	1.30	0.08–21.68	0.86
CHG, alcohol, and povidone-iodine	2	0.6	2	0.6	0	0.0			
Average temperature (°C), median (IQR)	36.0	(35.6, 36.4)	36.0	(35.6, 36.4)	35.1	(35.1, 35.1)	0.18	0.01–2.93	0.23
Minimum temperature(°C), median (IQR)	35.4	(34.8, 35.9)	35.4	(34.8, 35.9)	34.9	(34.9, 34.9)	0.44	0.03–7.06	0.56
Propofol (mg/kg), median(IQR)^a^	2.9	(2.0, 4.7)	2.9	(1.4, 3.8)	3.0	(2.1, 6.3)	1.00	0.91–1.10	0.96
ET sevoflurane, median(IQR)^a^	192	(102, 293)	192	(102, 298)	172	(116, 253)	1.00	0.996–1.003	0.78
ET isoflurane, median(IQR)^a^	0	(0, 0)	0	(0, 0)	0	(0, 0)	0.99	0.97–1.01	0.29
O2 flow, median(IQR)^a^	347	(272, 440)	347	(271, 451)	340	(278, 425)	1.00	0.996–1.004	0.99

*SSIs* surgical site infection, *IQR* interquartile range, *ASA* American Society of Anesthesiologists physical status classification, *GA* gestational age, *V-P* ventriculo-peritoneal, *EVD* external ventricular drain, *ETV* endoscopic third ventriculostomy, *V-A* ventriculo-atrial, *L-P* Lumbo-peritoneal, *SD* standard deviation, *ET* end tidal, *CHG* chlorhexidine gluconate

*Univariate analysis was performed

^a^The amount of milligram per kilogram of body weight was calculated for intravenous drugs, and the end tidal concentration per minute for volatile anesthetics or oxygen was summed up by the anesthesia duration (minutes)

Table 3 displays all available variables evaluated for potential association with SSI following EVD procedures. No variables in this analysis were significantly associated with SSIs. There was no SSI that occurred among patients undergoing ETV.

**Table 3:** Results of unadjusted logistic regression analyses of patient background and perioperative factors in patients underwent EVD procedures

	Total (***N*** = 72)		Case without SSI (***N*** = 67)		Case with SSI (***N*** = 5)		Unadjusted logistic regression		
	n	%	n	%	n	%	Odds ratio	95% CI	*p* -value
Female	42	58.3	41	61.2	1	20	0.18	0.02–1.66	0.13
Age(Month), median (IQR)	115	(30, 170)	107	(26, 172)	145	(118, 145)	1.00	0.99–1.02	0.58
Age									
< 1y	10	13.9	10	14.9	0	0.0	(base)		
1-2y	11	15.3	11	16.4	0	0.0			
3-10y	19	26.4	17	25.4	2	40.0	1.02	0.15–6.75	0.98
11-18y	29	40.3	26	38.8	3	60.0			
18y-	6	8.3	6	9.0	0	0.0			
Weight(kg), median (IQR)	32.2	(12.6, 53.3)	32.2	(12.6, 53.3)	34.8	(31, 35)	1.00	0.97–1.04	0.80
ASA Classification									
Class ≤ II	12	16.7	12	17.9	0	0.0			
Class ≥ III	63	87.5	58	86.6	5	100.0	3.75	0.58–24.16	0.16
Wound Class									1.00*
1: Clean	68	94.4	66	98.5	2	40.0			
2: Clean-Contaminated	1	1.4	1	1.5	0	0.0			
3: Contaminated	0	0.0	0	0.0	0	0.0			
4: Dirty/Infected	3	4.2	3	4.5	0	0.0			
Comorbidity									
Brain tumor	26	36.1	24	35.8	2	40.0	1.28	0.20–8.18	0.80
Cardiac disease	3	4.2	2	3.0	1	20.0	8.50	0.63–114.86	0.11
Gastrostomy status	13	18.1	12	17.9	1	20.0	1.21	0.12–11.79	0.87
Prematurity (GA < 37 week)	2	2.8	2	3.0	0	0.0			1.00*
Number of shunt revision, mean (SD)	1	(1, 3)	1	(1, 3)	0.5	(0, 3.5)	0.92	0.59–1.42	0.70
Emergent surgery	61	84.7	58	86.6	3	60.0	0.31	0.05–2.06	0.23
Duration of surgery(min), median(IQR)	51	(27, 74)	51	(37, 74)	38.5	(26, 51)	0.96	0.89–1.04	0.35
Prophylactic antibiotics	65	90.3	61	91.0	4	80.0	0.59	0.06–5.89	0.65
Cefazolin	55	76.4	51	76.1	4	80.0	1.49	0.16–14.19	0.73
Ampicillin	0	0.0	0	0.0	0	0.0			
Cefoxitin	0	0.0	0	0.0	0	0.0			
Ciprofloxacin	0	0.0	0	0.0	0	0.0			
Cefepime	0	0.0	0	0.0	0	0.0			
Ceftazidime	2	2.8	2	3.0	0	0.0			1.00*
Ceftriaxone	4	5.6	4	6.0	0	0.0			1.00*
Clindamycin	2	2.8	2	3.0	0	0.0			1.00*
Metronidazole	1	1.4	1	1.5	0	0.0			1.00*
Vancomycin	6	8.3	6	9.0	0	0.0			1.00*
Meropenem	0	0.0	0	0.0	0	0.0			
Surgeon *n* = 10	–	–	–	–	–	–			0.46*
Skin preparation agent									
None	11	15.3	10	14.9	1	20.0	(base)		
CHG-alcohol(Chloraprep™)	4	5.6	4	6.0	0	0.0	0.64	0.06–6.79	0.71
Povidone-iodine	50	69.4	47	70.1	3	60.0	1.11	0.06–20.49	0.94
Povidone-iodine and CHG-alcohol	0	0.0	0	0.0	0	0.0			
Povidone-iodine and alcohol	10	13.9	9	13.4	1	20.0			
CHG, alcohol, and povidone-iodine	0	0.0	0	0.0	0	0.0			
Average temperature (°C), median (IQR)	36.3	(35.6, 36.7)	36.3	(35.6, 36.7)	36.4	(36.4, 36.4)	1.39	0.11–18.0	0.80
Minimum temperature(°C), median (IQR)	35.5	(34.8, 36.2)	35.5	(34.8, 36.2)	35.6	(35.6, 35.6)	1.07	0.13–8.67	0.95
Propofol (mg/kg), median(IQR)	2.9	(1.4, 4.5)	2.9	(1.4, 4.4)	3.4	(2, 6.4)	1.04	0.97–1.12	0.28
ET sevoflurane, median(IQR)	91	(27, 191)	104	(32, 193)	21	(0, 69)	0.99	0.98–1.00	0.16
ET isoflurane, median(IQR)	0	(0, 19)	0	(0, 6)	0	(0, 34)	1.01	0.99–1.03	0.21
O2 flow, median(IQR)	280	(193, 343)	282	(199, 344)	128	(77, 215)	0.99	0.985–1.00	0.23

*SSIs* surgical site infection, *EVD* external ventricular drain, *IQR* interquartile range, *ASA* American Society of Anesthesiologists physical status classification, *GA* gestational age, *SD* standard deviation, *ET* end tidal, *CHG* chlorhexidine gluconate

*We used univariate analysis

Table 4 presents the CSF culture results for patients with SSIs. *Staphylococcus epidermidis* (14.3%) and *Cutibacterium acnes* (formerly *Propionibacterium acnes*) (14.3%) were detected most frequently. From the 19 positive CSF cultures, 14 unique bacterial and fungal species were identified.

**Table 4:** Date of SSIs onset, culture results and list of prophylactic antimicrobial agents by procedure

Procedure	Median interval (days)	SSI interval (POD)	Culture date (POD)	Culture resources	Culture result	Prophylactic antibiotics	SSI type
VP shunt	18.5	4	4	CSF	*Staphylococcus aureus*	cefepime/vancomycin	Organ/Space
		31	38	CSF peritoneal fluid shunt catheter tip		clindamycin	Organ/Space
		63	63	CSF	*Staphylococcus capitis*	cefazolin	Organ/Space
		11	11	CSF	*Staphylococcus capitis*	cefazolin	Organ/Space
		53	53	CSF	*Cutibacterium acnes*	clindamycin	Organ/Space
		29	30	Catheter Tip	*Enterococcus avium*	clindamycin	Organ/Space
		2	2	CSF	*Rothia aeriaStreptococcus viridans*	cefazolin	Organ/Space
		5	6	VP Shunt	*Corynebacterium coyleaeCutibacterium acnes*	cefazolin	Organ/Space
		15	15	CSF	*Serratia marcescens*	cefazolin	Organ/Space
		36	36	CSF	*Trichosporon asahii*	cefazolin	Organ/Space
		15	15	CSF		cefazolin	Organ/Space
		89	89	CSF, shunt tubing, shunt swab		cefazolin	Organ/Space
		10	10	CSF		cefazolin	Organ/Space
		22	22	CSF	*Cutibacterium acnes*	cefazolin	Organ/Space
EVD		20	21	CSF	*Stenotrophomonas maltophilia*	cefazolin	Organ/Space
		2	2	CSF	*Staphylococcus epidermidis*		Organ/Space
		9	9	CSF	*Staphylococcus epidermidis*	cefazolin	Organ/Space
		6	66	CSF	*Acinetobacter sp. Microbacterium Caulobacter segnis*	cefazolin	Organ/Space
		12	11	CSF	*Staphylococcus epidermidis*	cefazolin	Organ/Space

*SSIs* surgical site infection, *POD* Postoperative day, *V-P* ventriculo-peritoneal, *EVD* external ventricular drain, *CSF* cerebrospinal fluid

## Discussion

In this study, we investigated risk factors for pediatric SSIs after CSF diversion surgery at a single quaternary care pediatric center. We found that receipt of clindamycin for perioperative prophylaxis and comorbid cardiac disease were significantly associated with SSI in shunt surgery.

In our study, the incidence of SSI in combined CSF diversion surgery was 3.4%, while SSI rates for shunt surgery, EVD, and ETV were 4.3, 6.9, and 0%, respectively. EVD is a device placed to manage intracranial pressure when normal flow of CSF inside the brain is hindered. Because it is externalized, it is not surprising that EVD was associated with the highest infection rate among these procedure types. ETV is an important procedure for hydrocephalus that serves as an alternative to shunt device implantation. The success rate of ETV and its use in various ages and background diseases vary across reports [10]. ETV can be done with or without CPC. In our institution, CPC is generally done together with ETV. In general, the SSI rate of ETV is lower than that of a shunt device implantation procedure, but the post-ETV SSI rate depends on background and age [11]. In our institution, ETV was often performed for hydrocephalus due to congenital diseases in infancy, but it was also performed in cases of repeated shunting and brain tumor complications.

Research on modifiable perioperative risk factors and interventions to reduce the risk of shunt infection has been done for many years. Traditional risk factors include the duration of surgery [12], the experience of the surgeon [13], hair shaving [14], prophylactic systemic antibiotics [8, 15], antibiotic-impregnated sutures [16] and skin preparation [5, 6]. In our cohort, surgeon, duration of surgery, and skin preparation agent were not associated with SSIs. In addition, our institution routinely utilizes antibiotic-impregnated shunt tubing (Bactiseal tubing) [17, 18]. Receipt of clindamycin for prophylaxis was significantly associated with SSI. Cefazolin is the standard, first line prophylactic antibiotic choice for shunt operations, but other antibiotics are used when patients are thought to have penicillin or cephalosporin allergy. The spectrum of activity of clindamycin includes staphylococci, streptococci, pneumococci, and some anaerobic bacteria. However, it does not have activity against aerobic Gram negative bacteria. It is well known that unsubstantiated penicillin allergy labels are common in surgical patients. Up to 98% of patients labeled as allergic to penicillin can actually safely receive the antibiotic when tested [19]. Similarly, gastrointestinal upset is often listed as a reason for allergy to cephalosporins. In women with chorioamnionitis undergoing cesarean delivery, the use of cefazolin was associated with a lower incidence of postpartum infection than the use of clindamycin [20]. Although our study is from a single institution, this finding highlights the importance of obtaining a detailed history of antibiotic allergies and considering de-labeling strategies in order to maximize the number of patients who can successfully receive beta-lactam antibiotics for perioperative prophylaxis.

The association of cardiac diseases with shunt infection was previously described in a study of children who underwent shunt surgery within the first year of life [21]. Our study consisting of older children also demonstrated that comorbid cardiac disease was associated with an increased risk of shunt SSI. Cardiac diseases present among our patients with SSI included single ventricle disease with palliative repair [22] and valvulopathy with underlying connective tissue diseases. Although not directly related to infection, cardiac anomalies were associated with shunt failure in the prospective study of children from six Hydrocephalus Clinical Research Network Centers [23]. The authors suggested that this finding may be related to repeated hospitalizations and surgeries or immunodeficiency, but they did not formally analyze these exposures. For example, thymectomy is often performed to have better visualization in neonatal and infant cardiac surgeries [24], but it may have some immunological impact [25]. Immunological profiles for patients with a cardiac disease who present for CSF diversion procedures are not known, and could be a topic of future investigation.

The association between age and SSIs varies across studies. While some studies have identified younger age, particularly infancy, as a risk factor for SSI [26–29], other studies have demonstrated that age is not a risk factor for shunt failure or infection [30, 31]. Post- intraventricular hemorrhage (IVH) hydrocephalus secondary to prematurity occurs in infants, but older children may have different etiology of hydrocephalus including tumor-related hydrocephalus. In our cohort, age was not a risk factor. Most of the patients in our cohort were beyond infancy, and our results may not generalize to premature infants for that reason.

The risk of shunt failure or infection generally increases with each subsequent shunt replacement, resulting in a rising cumulative risk of SSIs [29, 32, 33]. However, in the present study, the number of shunt revisions was not a risk factor for SSIs. Previously, other investigators found that the presence of a gastrostomy tube was associated with infection risk in the larger study [32], but this did not substantially contribute to infections in this study. Some studies showed that lower gestational age (GA) at the time of procedures and prematurity were significant risk factors for infection after shunt surgery [7, 28]. In our study cohort, there was no incidence of SSIs in patients with a history of preterm birth, although a number of patients did not have detailed perinatal information available. Although it is difficult to determine the association between GA at birth and SSIs, GA was not a risk factor for SSI in our study. Further investigation is needed to clarify why our result differs from the previous reports.

Among the perioperative factors other than those mentioned above, there have been no reports that have examined the type and dose of anesthetics and the amount of oxygen administered in shunt surgery. Intraoperative factors such as oxygen dosage, and temperature control are known to play a role in SSI following adult general surgery [34–36]. The effect of volatile anesthetics on immune function have been reported previously [37–44]. In our previous work using a preclinical model, a long exposure (6-h) of volatile anesthetics was associated with increased infection, while a short exposure (2-h) was not [45]. In a study at our institution, we previously showed that higher volatile anesthetic dose was an independent risk factor for SSI in pediatric gastrointestinal surgery [46]. Anesthetic drugs and oxygen dosage also had no impact on the incidence of SSIs. The operative duration was less than 80 min for the groups with and without SSIs. Our result was in line with the finding in our preclinical study. In the present study, the median number of days to the date of SSI onset after surgery was 13.5 days, but more than 75% of the patients with SSI had a postoperative period of more than 30 days. In previous reports, the risk of infection was shown to be highest in the first 8 weeks after a shunt procedure, and the risk decreased substantially after 6 months [47]. Because of relatively late onset of SSI postoperatively and the short surgical duration, it is perhaps not surprising that the impact of intraoperative drugs and oxygen dose on immune function was less after CSF diversion surgery.

The most common bacteria detected in our cohort were *Staphylococcus epidermidis* and *Cutibacterium acnes*, generally consistent with previous reports [26, 27, 48, 49]. *Staphylococcus aureus* was detected in a smaller proportion of cases. While the effectiveness of antimicrobial-impregnated and -coated shunts (AIS) has been described [15, 49], there are concerns including the emergence of methicillin-resistant *Staphylococcus aureus* (MRSA) and resistant gram negative rods (GNR) with repeated use [48, 49]. Although we routinely use antimicrobial impregnated shunting, MRSA was not detected in our cohort.

Our study has several limitations. First, it was limited as a single-center study and was retrospective. Second, the low SSI rate and small sample size may impair statistical power to find associations.

In conclusion, we have shown that receipt of clindamycin as prophylaxis was significantly associated with SSI in children undergoing shunt surgery. Because unsubstantiated allergy labeling for penicillin and other antibiotics are common in surgical patients, efforts should be made to identify which patients can successfully receive a beta-lactam antibiotic for perioperative prophylaxis. Further research is needed to better define the mechanism by which cardiac disease is associated with SSI among patients undergoing neurosurgical shunt procedures.

## Data Availability

All the data and materials are available upon request.
